# Antimicrobial susceptibility testing of *Enterobacteriaceae*: determination of disk content and Kirby-Bauer breakpoint for ceftazidime/avibactam

**DOI:** 10.1186/s12866-019-1613-5

**Published:** 2019-11-01

**Authors:** Xianggui Yang, Dan Wang, Qin Zhou, Fang Nie, Hongfei Du, Xueli Pang, Yingzi Fan, Tingting Bai, Ying Xu

**Affiliations:** Department of Laboratory Medicine, the First Affiliated Hospital of Chengdu Medical College, 278 Baoguang Road, Xindu, Chengdu, Sichuan 610500 People’s Republic of China

**Keywords:** Ceftazidime/avibactam, Disk content, Kirby-Bauer breakpoints, *Enterobacteriaceae*

## Abstract

**Background:**

Detection of ceftazidime/avibactam (CAZ/AVI) antibacterial activity is absolutely vital with the rapid growth of carbapenem resistant *Enterobacteriaceae* (CRE). But now, there is no available automated antimicrobial susceptibility testing card for CAZ/AVI, so Kirby-Bauer has become an economical and practical method for detecting CAZ/AVI antibacterial activity against *Enterobacteriaceae*.

**Result:**

In this study, antimicrobial susceptibility testing of CAZ/AVI against 386 *Enterobacteriaceae* (188 *Klebsiella pneumoniae*, 122 *Escherichia coli*, 76 *Enterobacter cloacae*) isolated from clinical patients was performed by broth microdilution. Of the 386 strains, 54 extended spectrum β lactamases negative (ESBL(−)), 104 extended spectrum β lactamases positive (ESBL(+)), 228 CRE. 287 isolates were susceptible to CAZ/AVI and 99 isolates were resistant to CAZ/AVI. At the same time, to obtain optimal content avibactam (AVI) disk containing ceftazidime (30 μg), inhibition zone diameter of four kinds of ceftazidime (30 μg) disk containing different AVI content (0 μg, 10 μg, 25 μg, 50 μg) were tested by Kirby-Bauer method. The microdilution broth method interpretation was used as the standard to estimate susceptible or resistance and then coherence analysis was carried out between Kirby-Bauer and broth microdilution. The result shows the inhibition zone diameter of 30 μg/50 μg disk, susceptible isolates: 20.5 mm–31.5 mm, resistance isolates: 8.25 mm–21.5 mm. The inhibition zone diameter of 30 μg/25 μg disk, susceptible isolates: 19.7 mm–31.3 mm, resistance isolates: 6.5 mm–19.2 mm. The inhibition zone diameter of 30 μg/10 μg disk, susceptible isolates: 19.5 mm–31 mm, resistance isolates: 6.5 mm–11 mm. The inhibition zone diameter of ceftazidime (30 μg), susceptible isolates: 6.5 mm–27.5 mm, resistance isolates 6.5 mm.

**Conclusion:**

Our results show that 30 μg/50 μg, 30 μg/25 μg, 30 μg/10 μg CAZ/AVI disk have significant statistical differences to determinate CAZ/AVI antibacterial activity, but for 30 μg/50 μg disk, there has a cross section between susceptible isolates (minimum 20.5 mm) and resistance isolates (maximum 21.5 mm). For 30 μg/25 μg disk, it is hard to distinguish the difference between susceptible isolates (minimum 19.7 mm) and resistance isolates (maximum 19.2 mm), so 30 μg/10 μg CAZ/AVI disk is more conducive to determinate antibacterial activity.

## Background

Multidrug-resistant gram-negative bacteria, such as extended spectrum β lactamases (ESBL)-producing and carbapenemase-producing *Enterobacteriaceae*, are rapidly prevalent worldwide and cause huge medical burden and mortality from these organism infections [1, 2]. Carbapenem antibiotics have been hailed as the most effective drug for the treatment of ESBL-producing *Enterobacteriaceae* [3], but for now, the numbers of carbapenem-resistant *Enterobacteriaceae* (CRE) have increased dramatically [4–6], so it is imminent to develop new antibiotics against carbapenemase-producing *Enterobacteriaceae* all around the world. Avibactam (AVI) is a potent, novel diazabicyclooctane β-lactamase inhibitor with in vitro activity against classes A, C and some class D β-lactamases [7, 8]. When used in combination with ceftazidime (CAZ), AVI restores the activity of CAZ against Gram-negative organisms producing these carbapenemases, as well as any co-carried ESBLs [9, 10]. Unfortunately, up to now, there is no available automated antimicrobial susceptibility testing card for ceftazidime/avibactam (CAZ/AVI), so Kirby-Bauer has become an economical and practical method for detection of CAZ/AVI activity against *Enterobacteriaceae*. Here, we aimed to evaluate the effect of different content AVI on CAZ/AVI activity against *Enterobacteriaceae*, to obtain optimal content AVI and to determine the breakpoint of CAZ/AVI, so as to provide guidance for clinical use of CAZ/AVI.

## Results

### Resistant type of strains in this study

Table 1 shows 386 strains resistant type, 188 *Klebsiella pneumonia* (18 ESBL(−)(4.7%), 37 ESBL(+)(9.6%), 133 CRE (34.5%)), 122 *Escherichia coli*, (27 ESBL(−)(7.0%), 37 ESBL(+)(9.6%), 58 CRE (15.0%)), 76 *Enterobacter cloacae* (9 ESBL(−)(2.3%), 30 ESBL(+)(7.8%), 37 CRE (9.6%)).

**Table 1:** Resistant type of strains in this study

Resistant type	ESBL(−) ^a^			ESBL(+) ^a^			CRE ^a^		
stains	KP	ECO	EC	KP	ECO	EC	KP	ECO	EC
No. tested	18	27	9	37	37	30	133	58	37
%	4.7	7.0	2.3	9.6	9.6	7.8	34.5	15.0	9.6

KP: *Klebsiella pneumonia*. ECO: *Escherichia coli*. EC: *Enterobacter cloacae*. ESBL: extended-spectrum beta-lactamase. CRE: Carbapenem-resistant *Enterobacteriaceae*

^a^Criteria as published by the Clinical and Laboratory Standards Institute (Clinical & Laboratory Standards Institute, M100, 28th Edition, 2018)

### Broth microdilution MICs for CAZ/AVI against Enterobacteriaceae

Table 2 indicates the isolates broth microdilution minimum inhibitory concentration (MIC) for CAZ/AVI. Using the Clinical & Laboratory Standards Institute (CLSI) breakpoint (CLSI, M100, 28th Edition, 2018) for CAZ/AVI, susceptible: MIC≤8/4 μg/ml, resistant: MIC≥16/4 μg/ml, percentage susceptibility for CAZ/AVI: *Klebsiella pneumonia* 85.6% (161/188), *Escherichia coli* 66.4% (81/122), *Enterobacter cloacae* 59.2% (45/76). Together with Table 1 results, we find that the CAZ/AVI resistant strains are all carbapenem-resistant *Enterobacteriaceae*.

**Table 2:** MIC distribution of ceftazidime/avibactam

Species	Susceptible ^a^		Resistant ^a^	S%
	MIC≤0.5	0.5<MIC≤8	MIC≥16	
KP (No. tested)	103	58	27	85.6%
ECO (No.tested)	37	44	41	66.4%
EC (No. tested)	24	21	31	59.2%

KP: *Klebsiella pneumonia*. ECO: *Escherichia coli*. EC: *Enterobacter cloacae*

a: Criteria as published by the Clinical and Laboratory Standards Institute (Clinical & Laboratory Standards Institute, M100, 28th Edition, 2018)

### Disk diffusion inhibition zones analysis

**Fig. 1:**
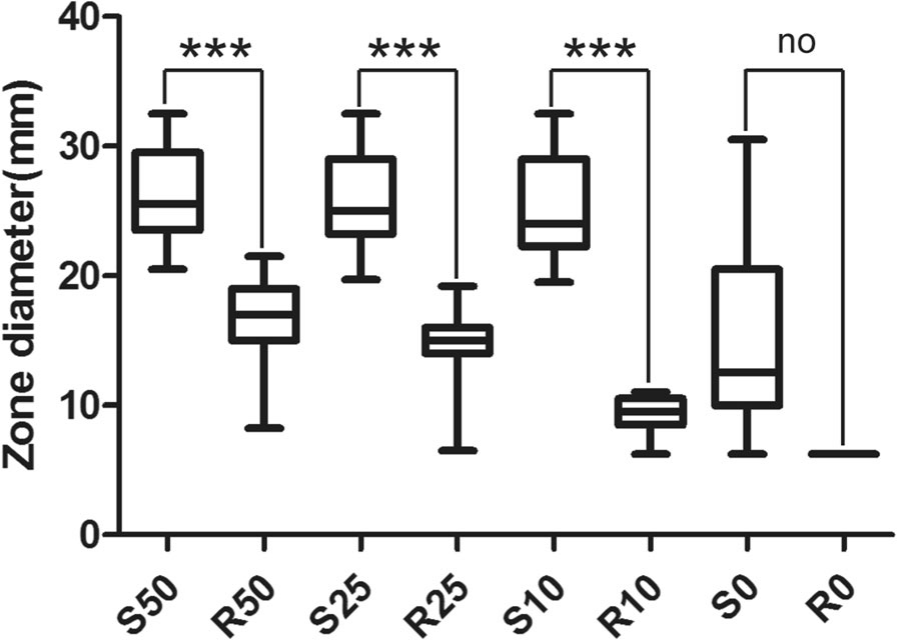
The disk diffusion inhibition zones diameter. S: susceptible isolates for ceftazidime/avibactam. R: Resistant isolates for ceftazidime/avibactam. 50: ceftazidime/avibactam disk content 30 μg /50 μg, 25: ceftazidime/avibactam disk content 30 μg /25 μg, 10: ceftazidime/avibactam disk content 30 μg /10 μg, 0: ceftazidime disk content 30 μg, ***P < 0.001 indicate highly statistically significant differences. no: no statistical analysis

Figure 1 shows the disk diffusion inhibition zone diameter, for CAZ/AVI (30 μg/50 μg) disk, susceptible isolates inhibition zone diameter 20.5 mm–31.5 mm, resistant isolates inhibition zone diameter 8.25 mm–21.5 mm, for CAZ/AVI (30 μg/25 μg) disk, susceptible isolates inhibition zone diameter 19.7 mm–31.3 mm, resistant isolates inhibition zone diameter 6.5 mm–19.2 mm, for CAZ/AVI (30 μg/10 μg) disk, susceptible isolates inhibition zone diameter 19.5 mm–31 mm, resistant isolates inhibition zone diameter 6.5 mm–11 mm. From our result, for CAZ/AVI (30 μg/50 μg) disk, the inhibition zone diameter has a cross section between susceptible isolates (minimum 20.5 mm) and resistance isolates (maximum 21.5 mm). Although there is no cross section between susceptible isolates (minimum 19.7 mm) and resistance isolates (maximum 19.2 mm) in inhibition zone diameter of CAZ/AVI (30 μg/25 μg) disk, it is hard to distinguish the difference between minimum (19.7 mm) and maximum (19.2 mm). While, the 30 μg/10 μg CAZ/AVI disk inhibition zone diameter has significantly difference between susceptible isolates and resistant isolates. To further stratify the inhibition zone diameter distribution, we analysis the inhibition zone diameter range of the isolates with MIC, as shows in the Additional file 1: Table S1, when the MIC value ≥256, the inhibition zone diameter is 6.5 mm (the diameter of disk). When the MIC value ≤0.25, inhibition zone diameter: 25.4 mm–31 mm. When 0.5 ≤ MIC≤8, inhibition zone diameter falls 19.3 mm–27.6 mm. When the MIC is between 16 and 128, the diameter of inhibition zone is fixed at 6.5 mm–11 mm.

### Recommended ceftazidime/avibactam disk content and breakpoint for Enterobacteriaceae, and sensitivity and specificity

The Table 3 indicate our recommended CAZ/AVI disk content: CAZ 30 μg, AVI 10 μg. CAZ/AVI disk breakpoint: susceptible≥20 mm, resistant≤11 mm, 11 mm–20 mm interpret as susceptible-dose dependent or intermediate. As reported in Additional file 2: Table S2, the inhibition zone diameter cut-off we recommended is more conducive to evaluate the antibacterial activity of CAZ/AVI against *Enterobacteriaceae* and achieves perfect specificity (100%) and excellent sensitivity (*Klebsiella pneumonia*:96.9%, *Escherichia coli*:97.5%, *Enterobacter cloacae*:95.6%, overall:96.9%).

**Table 3:** Recommended ceftazidime/avibactam disk information for *Enterobacteriaceae*

antibiotic	Disk content (μg)	inhibition zone diameter cut-off (mm)	
		Susceptible	Resistant
ceftazidime/avibactam	30/10	≥20	≤11

## Discussion

Increasing carbapenem-resistant *Enterobacteriaceae* (CRE) have drawn great attention because of their broad resistance spectra and outbreak epidemics [11, 12], as well as have shown a high potential of rapid disseminations [13]. In 2012, a study reported that 3% of ICU patients in Chicago were infected with CRE, among ICU medical staff, the colonization rate of CRE was as high as 30% [14]. In 2016, seven children were infected with non-clonal CRE in pediatric hospital in Mexico [15]. Between 2005 and 2010, carbapenem-resistant *Klebsiella pneumoniae* invasive infections occurred successively in Greece, Italy, Hungary and Cyprus [16]. European infection control experts reported the interregional transmission or epidemic of CRE in 13/38 countries in 2015, compared with in 6/38 countries in 2013 [17]. Between 2004 and 2013, CRE has gradually increased in medical centers and major medical teaching centers, and prevalence rates of CRE isolated from ICU rose from 3.7% in 2008 to 15.3% in 2017 in Taiwan [18]. In addition, in ICU, the isolation rate of CRE *Escherichia coli* increased from 1.2% in 2008 to 4.0% in 2017 in medical centers, while the CRE isolation rate in major teaching centers increased from 1.0% in 2008 to 2.8% in 2017 [18]. Because of high mortality (> 30%), infection caused by CRE has become the major worrying health event all around the world [19–21]. In 2017, the World Health Organization listed CRE as the first “critical priority pathogens” [22]. These scattered findings in different parts of the world emphasize the fact that the development of new antibiotics against CRE is imminent. Currently, CRE treatment mainly depends on older agents, such as polymyxins, fosfomycin, tigecycline and aminoglycosides, which have been rarely used due to efficacy and/or toxicity concerns [23]. Recently, several drugs were tested to gauge their effectiveness against CRE infections, such as ceftazidime/avibactam (CAZ/AVI), CAZ/AVI is a fixed-dose combination drug containing an antibiotic-third generation cephalosporin ceftazidime and a novel non-β-lactam β-lactamase inhibitor avibactam [24, 25]. Previously approved β-lactamase inhibitors such as tazobactam and clavulanic acid do not inhibit important classes of β-lactamases, including *Klebsiella pneumoniae* carbapenemases (KPCs), New Delhi metallo-β-lactamase 1 (NDM-1), and AmpC-type β-lactamases [26]. Avibactam can inhibit KPCs, AmpC, and some Class D β-lactamases [25]. Therefore, CAZ/AVI has preferable antibacterial activity against the majority CRE. Unfortunately, there is no available automated antimicrobial susceptibility testing card for CAZ/AVI, so Kirby-Bauer has become an economical and practical method to evaluate CAZ/AVI activity against *Enterobacteriaceae*. Here, we estimate the disk diffusion of CAZ/AVI cut-off zones and disk content.

In this study, from two different regions of China (Chengdu and Chongqing), a total of 386 clinical isolates of *Enterobacteriaceae* isolated from patient were collected, 54 ESBL(−) isolates (27 *Escherichia coli*, 18 *Klebsiella pneumonia*, 9 *Enterobacter cloacae*), 104 ESBL(+) isolates (37 *Escherichia coli*, 37 *Klebsiella pneumonia*, 30 *Enterobacter cloacae*), 228 CRE isolates (58 *Escherichia coli*, 133 *Klebsiella pneumonia*, 37 *Enterobacter cloacae*). Of which, according to CLSI CAZ/AVI breakpoint for *Enterobacteriaceae* (susceptible MIC≤8/4, resistant MIC≥16/4), 27 *Klebsiella pneumonia,* 41 *Escherichia coli,* and 31 *Enterobacter cloacae* are resistant to CAZ/AVI (all resistant isolates are CRE). At present, various resistance mechanisms of CAZ/AVI have been reported, including substitutions of specific amino acid in some beta-lactamases such as KPCs, CTX-M-14 [27–29], OXA-2 duplication [30], and loop deletion in AmpC beta-lactamase [31]. In addition, mutations in membrane porin [29, 32, 33], enhanced efflux pump activity [29], and sustained high expression of certain beta lactamases can lead to CAZ/AVI resistance [32]. So in the follow-up study, we will explore the specific resistance mechanisms. Here, our data shows resistant percentages of *Klebsiella pneumonia*, *Escherichia coli,* and *Enterobacter cloacae* are 20.3% (27/133), 70.7% (41/58), and 83.8% (31/37) for CAZ/AVI, respectively, which inconsistent with other researchers [34–36]. The follow two causes may be account for the difference. Firstly, our isolates collected according to random selection within group (ESBL negative isolates collected from ESBL negative group, ESBL positive isolates collected from ESBL positive group, CRE isolates collected from CRE group). Second, the last 2 years, new CRE organisms increasing rapidly, such as NDM-1-producing *Enterobacteriaceae*, which can not be inhibited by AVI [37].

Although CAZ/AVI had a good antibacterial activity against multidrug-resistant Gram-negative bacteria, it was not until 2017 that there was a cut off CAZ/AVI against *Enterobacteriaceae* in the report of European Committee on Antimicrobial Susceptibility Testing (EUCAST, document. 2017). In 2017, EUCAST report CAZ/AVI inhibition zone diameter cut off and MIC breakpoint, for Kirby-Bauer method (disk content 10 μg /4 μg), susceptible (inhibition zone diameter >13 mm) and resistant (inhibition zone diameter <13 mm), for microdilution broth method (a fixed concentration 4 μg/ml), susceptible (MIC<8/4 μg /ml) and resistant (MIC>8/4 μg /ml). CLSI reported CAZ/AVI inhibition zone diameter cut off and MIC breakpoint in 2018 edition, for Kirby-Bauer method (disk content 30 μg /20 μg), susceptible (inhibition zone diameter ≥ 21 mm) and resistant (inhibition zone diameter ≤ 20 mm), for microdilution broth method (a fixed concentration 4 μg /ml), susceptible (MIC≤8/4 μg /ml) and resistant (MIC≥16/4 μg /ml). Unfortunately, there are still no commercial kits for determining CAZ/AVI antibacterial activity, so we believe that Kirby-Bauer is an economical method for CAZ/AVI. In this study, in order to screen optimal content AVI disk containing ceftazidime (30 μg), inhibition zone diameters of four kinds of ceftazidime (30 μg) disk containing different AVI content (0 μg, 10 μg, 25 μg, 50 μg) against 122 *Escherichia coli*, 188 *Klebsiella pneumonia* and 76 *Enterobacter cloacae* were tested, the CLSI broth microdilution method interpretation was used as the standard to estimate activity.

The result shows that 30 μg/50 μg, 30 μg/25 μg, 30 μg/10 μg CAZ/AVI disk have significant statistical differences and are conducive to determinate antibacterial activity (Fig. 1). Furthermore, disk diffusion breakpoint of CAZ/AVI (30 μg/10 μg) we recommended for *Enterobacteriaceae* (susceptible ≥20 mm and resistant ≤11 mm) achieves practicable sensitivity and specificity, as shows in Additional file 2: Table S2, for sensitivity (*Klebsiella pneumonia*:96.9%, *Escherichia coli*:97.5%, *Enterobacter cloacae*:95.6%, overall:96.9%) and 100% specificity.

Compare with CLSI breakpoint for CAZ-AVI disk (CAZ-AVI:30 μg/20 μg, susceptible≥21 mm, resistant≤20 mm), the 30 μg/10 μg CAZ/AVI disk inhibition zone diameter is a more simple and effective choice to evaluate CAZ/AVI activity (susceptible ≥20 mm and resistance≤11 mm).

## Conclusions

In conclusion, our study indicates that 30 μg/10 μg CAZ/AVI disk is a more feasible choice to evaluate CAZ/AVI activity against *Enterobacteriaceae*, and disk diffusion breakpoint CAZ/AVI (30 μg/10 μg) we recommended for *Enterobacteriaceae* (susceptible ≥20 mm and resistant ≤11 mm) has excellent sensitivity and specificity. Those data will provide a rapid and accurate detection of CAZ/AVI activity against the major *Enterobacteriaceae* and is conducive to provide more effective guidance for treatment of infectious diseases.

## Methods

### Bacterial strains

A total of 386 *Enterobacteriaceae* isolates (188 *Klebsiella pneumoniae*, 122 *Escherichia coli*, 76 *Enterobacter cloacae*) were collected according to random selection within group (ESBL negative isolates were random collected from ESBL negative group, ESBL positive isolates were random collected from ESBL positive group, CRE isolates were random collected from CRE group) from two different regions of China (Chengdu and Chongqing), all microorganisms were isolated from patient specimens (such as Urine, blood, sputum and secretion) during treatment, and then those isolates have to be preserved for scientific research. All strains were identified by GN card (bioMerieux, Durham, NC, USA) and ID32 GN (bioMerieux, Durham, NC, USA).

### Extended-spectrum β-lactamases experiment

Tests for Extended-Spectrum β-Lactamases was carried out in accordance with Clinical & Laboratory Standards Institute (CLSI, M100, 28th Edition, 2018), in brief, the disk ceftazidime 30 μg (BIO-KONT, Wenzhou, China), ceftazidime-clavulanate 30/10 μg (BIO-KONT, Wenzhou, China), cefotaxime 30 μg (BIO-KONT, Wenzhou, China) and cefotaxime-clavulanate 30/10 μg (BIO-KONT, Wenzhou, China) (Testing necessitates using both cefotaxime and ceftazidime, alone and in combination with clavulanate) was used to test inhibition zone diameter with disk diffusion method. A ≥ 5 mm increase in the inhibition zone diameter for either antimicrobial agent tested in combination with clavulanate vs the inhibition zone diameter of the agent when tested alone is regarded as ESBL positive (ESBL(+)), otherwise ESBL negative (ESBL(−)), *K. pneumoniae* ATCC® 700603(BIO-KONT, Wenzhou, China) and *E. coli* ATCC® 25922(BIO-KONT, Wenzhou, China) was used as positive control and negative control, respectively.

### CRE experiment

Minimum inhibitory concentration (MIC) of ertapenem, imipenem and meropenem were obtained with broth microdilution, those isolates of which resistant to one or more carbapenems (ertapenem, imipenem and meropenem) using the current MIC breakpoints (CLSI, M100, 28th Edition, 2018) were defined as carbapenem-resistant *Enterobacteriaceae* (CRE).

### CAZ/AVI disks preparation

CAZ (30μg) disk is purchased from Wenzhou Kangtai Biological Company (BIO-KONT, Wenzhou, China) and prepared in regulated good manufacturing practice conditions, AVI was obtained commercially (MedChemExpress. New Jersey. USA) and dissolved in water. CAZ/AVI disks were prepared as follows: 30 μg/0 μg disk: CAZ (30 μg) + 5ul H_2_O, 30 μg/10 μg disk: CAZ (30 μg) + 5ul AVI (2 mg/ml), 30 μg/25 μg disk: CAZ (30 μg) + 5ul AVI (5 mg/ml), 30 μg/50 μg disk: CAZ (30 μg) + 5ul AVI (10 mg/ml), those disks were dried naturally at room temperature and immediately used in the experiment. At the same time, *E. coli* ATCC 25922 and 35,218, and *Klebsiella pneumoniae* ATCC 700603 were used for quality control organisms.

### Ceftazidime/avibactam antimicrobial susceptibility test

Ceftazidime/avibactam MIC were tested by broth microdilution according to CLSI guideline (M100, 28th Edition, 2018) using a fixed concentration of avibactam (MedChemExpress. New Jersey. USA) 4 μg/ml. Each isolate was tested in duplicate; a third replicate was necessary if there was disagreement between the first two broth microdilution results. For Kirby-Bauer method, plates were incubated at 35 °C and read after 16–20 h incubation, ceftazidime (30 μg) disk containing different avibactam content (0 μg, 10 μg, 25 μg, 50 μg) were used for disk diffusion test, k diffusion tests were performed in triplicate in parallel with broth microdilution, the mean value of the three inhibition zone diameter is used for statistical analysis. *Escherichia coli* ATCC 25922 and *Escherichia coli* ATCC 32518 were used for quality control. Isolates were considered to be susceptible to CAZ/AVI when MICs were ≤ 8/4 mg/L (CLSI, M100, 28th Edition, 2018).

### Statistical analysis

T-test was employed to assess the statistical significance of differences intra-group comparisons using GraphPad Prism 5 software (GraphPad Software, Inc., La Jolla, CA, USA). Differences were considered statistically significant at *p* < 0.01.

## Supplementary information

**Additional file 1: Table S1.** The zone diameter range with MIC.

**Additional file 2: Table S2.** The sensitivity and specificity of 30 μg/10 μg CAZ/AVI disk.

## Data Availability

All authors decided the datasets used and analyzed in this study are available from the corresponding author upon reasonable request through e-mail. e-mail:yingxu@cmc.edu.cn

## Abbreviations

AVI: avibactam
CAZ: ceftazidime
CAZ/AVI: ceftazidime/avibactam
CLSI: Clinical & Laboratory Standards Institute
CRE: carbapenem resistant *Enterobacteriaceae*
ESBL: Extended spectrum β lactamases
ESBL(−): Extended spectrum β lactamases negative
ESBL(+): Extended spectrum β lactamases positive
EUCAST: European Committee on Antimicrobial Susceptibility Testing
MIC: Minimum inhibitory concentration
