# Supplementary material for: Antimicrobial susceptibility testing of Enterobacteriaceae: determination of disk content and Kirby-Bauer breakpoint for ceftazidime/avibactam
Source: BMC Microbiol. 2019 Nov 1;19:240. doi: 10.1186/s12866-019-1613-5 (PMC6824082; doi:10.1186/s12866-019-1613-5)
Supplement: Supplementary file 1 — Additional file 1: Table S1. The zone diameter range with MIC. [file 12866_2019_1613_MOESM1_ESM.docx]

**Table S1** The zone diameter range with MIC

| CAZ/AVI MIC (µg/ml) | CAZ/AVI（30µg/10µg）inhibition zone diameter range（mm） | | |
| --- | --- | --- | --- |
|  | KP | ECO | EC |
| MIC≤0.25 | 26.5-31 | 27.3-30.5 | 25.4-30.2 |
| 0.5≤MIC≤2 | 20.9-25.2 | 21.5-26.3 | 20.5-27.6 |
| 4≤MIC≤8 | 20.5-23.5 | 19.3-22.7 | 20.2-23.5 |
| 16≤MIC≤32 | 9.4-11 | 9.2-10.5 | 8.4-10.6 |
| 64≤MIC≤128 | 6.5-9.8 | 6.5-9.3 | 6.5-10.2 |
| MIC≥256 | 6.5 | 6.5 | 6.5 |

KP: *Klebsiella pneumonia*. ECO: *Escherichia coli*. EC: *Enterobacter cloacae*. MIC: Minimum inhibitory concentration.
