# Supplementary material for: Antimicrobial susceptibility testing of Enterobacteriaceae: determination of disk content and Kirby-Bauer breakpoint for ceftazidime/avibactam
Source: BMC Microbiol. 2019 Nov 1;19:240. doi: 10.1186/s12866-019-1613-5 (PMC6824082; doi:10.1186/s12866-019-1613-5)
Supplement: Supplementary file 2 — Additional file 2: Table S2. The sensitivity and specificity of 30 μg/10 μg CAZ/AVI disk. [file 12866_2019_1613_MOESM2_ESM.docx]

**Table S2** The sensitivity and specificity of 30µg/10µg CAZ/AVI disk

| Kirby-Bauer method | broth dilution method（golden standard） | | | |
| --- | --- | --- | --- | --- |
|  | susceptible(MIC≤8μg/ml) | | resistance（MIC≥16μg/ml） | |
| Susceptible  (IZD≥20mm) | TP | KP = 156 | FP | KP = 0 |
|  |  | ECO = 79 |  | ECO = 0 |
|  |  | EC = 43 |  | EC = 0 |
| Resistance (IZD≤11mm) | FN | KP=5 * | TN | KP = 27 |
|  |  | ECO=2* |  | ECO = 41 |
|  |  | EC=2* |  | EC = 31 |
| sensitivity | KP | TP/(TP+FN)=156/(156+5)=96.9% | | |
|  | ECO | TP/(TP+FN)=79/(79+2)=97.5% | | |
|  | EC | TP/(TP+FN)=43/(43+2)=95.6% | | |
|  | overall | TP/(TP+FN)=278/(278+9)=96.9% | | |
| specificity | KP | TN/(FP+TN)=27/(27+0)=100% | | |
|  | ECO | TN/(FP+TN)=41/(41+0)=100% | | |
|  | EC | TN/(FP+TN)=31/(31+0)=100% | | |
|  | overall | TN/(FP+TN)=99/(99+0)=100% | | |

KP: *Klebsiella pneumonia*. ECO: *Escherichia coli*. EC: *Enterobacter cloacae*. MIC: Minimum inhibitory concentration. IZD: inhibition zone diameter. TP: true positive. FP: false positive. FN: false negative. TN: true negative. *: The isolates MIC≤8μg/ml for CAZ/AVI, but the inhibition zone diameter (30μg/10μg CAZ/AVI disk) falls between 11mm and 20mm.
